# ReS_2_ Nanosheets with In Situ Formed Sulfur Vacancies for Efficient and Highly Selective Photocatalytic CO_2_ Reduction

**DOI:** 10.1002/smsc.202000052

**Published:** 2021-01-15

**Authors:** Yanzhao Zhang, Dazhi Yao, Bingquan Xia, Haolan Xu, Youhong Tang, Kenneth Davey, Jingrun Ran, Shi-Zhang Qiao

**Affiliations:** ^1^ School of Chemical Engineering and Advanced Materials The University of Adelaide Adelaide SA 5005 Australia; ^2^ Future Industries Institute University of South Australia Adelaide SA 5095 Australia; ^3^ Center for Nanoscale Science and Technology School of Computer Science Engineering, and Mathematics Flinders University Adelaide SA 5042 Australia

**Keywords:** CO_2_ photoreduction, heterojunctions, ReS_2_, sulfur vacancies, transition metal dichalcogenides

## Abstract

Artificial photosynthesis can provide valuable fuels and positively impact greenhouse effects, via transforming carbon dioxide (CO_2_) and water (H_2_O) into hydrocarbons using semiconductor‐based photocatalysts. However, the inefficient charge‐carrier dissociation and transportation as well as the lack of surface active sites are two major drawbacks to boosting their activity and selectivity in photocatalytic CO_2_ reduction. Recently, ReS_2_ has received tremendous attention in the photocatalysis area due to its intriguing physicochemical properties. Nevertheless, the application of ReS_2_ in photocatalytic CO_2_ reduction is scarcely covered. Herein, a heterojunction formed between ReS_2_ nanosheets and CdS nanoparticles is reported, achieving an apparently raised CO production of 7.1 μmol g^−1^ and high selectivity of 93.4%. The as‐prepared ReS_2_/CdS heterojunction exhibits strengthened visible‐light absorption, high‐efficiency electron–hole pair separation/transfer, and increased adsorption/activation/reduction of CO_2_ on in situ created sulfur vacancies of ReS_2_, thus all favoring CO_2_ photoreduction. These are corroborated by advanced characterization techniques, e.g., synchrotron‐based X‐ray absorption near‐edge structure, and density functional theory–based computations. The findings will be of broad interest in practical design and fabrication of surface active sites and semiconductor heterojunctions for applications in catalysis, electronics, and optoelectronics.

## Graphical abstract

Herein, a heterojunction formed between ReS_2_ nanosheets and CdS nanoparticles exhibits enhanced photocatalytic CO production together with excellent CO production selectivity. Advanced characterization, e.g., synchrotron‐based X‐ray absorption near‐edge structure, and theoretical computations disclose that the pronounced electronic coupling between ReS_2_ and CdS and in situ generated sulfur vacancies contribute to the apparently improved light‐induced CO_2_‐to‐CO conversion performance.

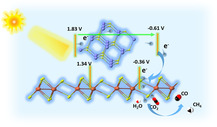

## 1 Introduction

CO_2_ emission leads to severe global greenhouse effect. Therefore, various strategies have been developed to relieve this process, including CO_2_ fixation and cyclic utilization.^[^ ^1^, ^2^, ^3^, ^4^ ^]^ Meanwhile, the application of clean and renewable energy to fix CO_2_ can not only promote the carbon cycle but also relieve the global energy crisis. Semiconductor‐based photocatalytic carbon dioxide (CO_2_) conversion represents a carbon‐neutral and sustainable strategy to generate fuels and chemicals using renewable and clean solar energy.^[^ ^1^, ^2^, ^3^, ^4^, ^5^ ^]^ This process is fundamentally impacted by three steps: 1) light absorption to excite semiconductor‐based photocatalysts, 2) photogenerated electron–hole pair separation and transfer efficiency, and 3) redox reactions on the surface of the photocatalyst.^[^ ^6^, ^7^ ^]^ To improve photocatalytic CO_2_ reduction, researchers have generally focused on approaches including introducing defects in the crystal lattice,^[^ ^8^, ^9^, ^10^, ^11^, ^12^, ^13^ ^]^ loading metal cocatalysts,^[^ ^14^, ^15^ ^]^ exposing highly active facets,^[^ ^16^, ^17^ ^]^ and fabricating heterojunctions.^[^ ^7^, ^18^ ^]^ Among these, forming heterojunctions in composites is deemed as an effective strategy due to efficient suppression of charge carrier recombination and highly promoted migration efficiency.^[^ ^19^, ^20^ ^]^ Some cocatalysts broaden the light absorption spectrum to utilize long‐wavelength light.^[^ ^21^, ^22^ ^]^ However, CO_2_ molecules are highly thermodynamically stable with a bond energy of 750 kJ mol^−1^ for C—O.^[^ ^6^ ^]^ This implies that the dissociation of CO_2_ requires high energy via conventional methods.^[^ ^6^ ^]^ The activation of CO_2_ molecules on the surface of photocatalysts relies on various active sites, e.g., functional groups, frustrated Lewis pairs, single atoms and vacancies. These possess an affinity for CO_2_ and water (H_2_O) adsorption and activation. These are however rare on the perfect surfaces of photocatalysts. The deliberate creation of active sites on the surfaces of photocatalysts is therefore a major research approach to practically realize effective photocatalysis.^[^ ^6^ ^]^

Transition metal dichalcogenides (TMDs) have received significant attention in catalysis, rechargeable batteries, and sensing devices.^[^ ^23^, ^24^, ^25^, ^26^, ^27^, ^28^ ^]^ For example, Zhou et al. reported that FeS ultrathin nanosheets on a carbon fiber cloth achieved highly efficient hydrogen evolution due to the phase transition triggered by illumination at room temperature.^[^ ^29^ ^]^ Similarly, Fu et al. prepared MoReS_3_ with a layered structure, a new type of TMDs, and it showed excellent hydrogen evolution in electrocatalysis.^[^ ^30^ ^]^ As a new type of TMDs, ReS_2_ has been studied in photocatalytic hydrogen evolution (PHE), both experimentally and theoretically.^[^ ^31^, ^32^, ^33^, ^34^, ^35^, ^36^, ^37^, ^38^ ^]^ Zhang et al.^[^ ^34^ ^]^ reported that ReS_2_ exhibited significant performance in PHE, a two‐electron catalytic reaction. This is because free electrons are captured by tightly bound excitons to form trions that constrain recombination and thereby promote performance. The corresponding computation indicates that the adsorption energy of the H atoms exhibits a small absolute value (−0.5 eV), revealing the highly efficient PHE performance. With a narrow bandgap of 1.7 eV, ReS_2_ possesses strong absorption in the visible‐light range, thus benefiting photocatalytic activity. In addition, ReS_2_ exhibits an automatic transition of hydrophobicity–hydrophilicity before and following visible‐light illumination.^[^ ^35^ ^]^ This is because defects on its surface change the adsorption configuration of H_2_O and oxygen (O_2_) to form hydroxyl groups leading to the transmission of hydrophilicity.^[^ ^35^ ^]^ This adsorption configuration impacts adsorption of CO_2_ and H_2_O in CO_2_ photoreduction. Zhou et al.^[^ ^36^ ^]^ reported that Re sites can absorb hydrogen as the intermediate for CO_2_ hydrogenation. Findings suggest ReS_2_ nanosheets (NSs) are a potential photocatalyst for CO_2_ reduction. A drawback however with most TMDs is that the reactive sites are located at the edge of the NSs, because of the hanging bond created by breakage of the bond between the transmitting metal and sulfur. These limited active sites on the edge and inert surface elsewhere are a significant impediment to applying TMDs as photocatalysts. Therefore, the activation of the inert surface through the introduction of defects on ReS_2_ NSs is regarded as an effective strategy to boost their photocatalytic performance.

Herein, we report a simple self‐assembly approach to synthesize a heterojunction composed of ReS_2_ NSs and CdS nanoparticles (NPs) for photocatalytic CO_2_ reduction. The optimized ReS_2_/CdS heterostructure exhibits a boosted photocatalytic CO_2_‐to‐CO conversion activity of 7.1 μmol g^−1^ accompanied by a high selectivity of 93.4%. The enhanced performance is aroused by high‐efficiency interfacial charge transfer between ReS_2_ and CdS as well as in situ formed sulfur vacancies on the ReS_2_ surface. Results from advanced characterizations, e.g., synchrotron‐based X‐ray absorption near‐edge structure (XANES), together with X‐ray photoelectron spectroscopy (XPS), confirm the strong electronic coupling between ReS_2_ and CdS. Density functional theory (DFT)‐based computations highlight the adsorption and activation of CO_2_ on the ReS_2_ surface with in situ formed sulfur vacancies based on electron transfer and change in C—O bond length and angle. Our work will be of immediate practical interest to a wide range of researchers for the design and synthesis of nanostructured materials in the field of energy conversion and storage.

## 2 Results and Discussion

ReS_2_ NSs were prepared by exfoliating commercial bulk ReS_2_ under ultrasonication in deionized water, and CdS NPs were fabricated by a hydrothermal method. Then, the heterojunctions of ReS_2_ and CdS were synthesized by physical mixing. The as‐prepared samples were denoted as CdS, CR4, CR8 and CR12, respectively, according to the added volumes of ReS_2_ NSs suspension (0, 4, 8 and 12 mL, respectively). The X‐ray diffraction (XRD) patterns of all the as‐prepared samples are shown in Figure S1, Supporting Information. The pattern displayed is ascribed to cubic‐phase CdS (PDF #10‐0454). A weak peak at 14.6° is attributed to the presence of ReS_2_ NSs. The intensity of this peak is enhanced with increasing content of ReS_2_ in the heterojunction. No apparent alteration of peak positions and intensities is observed for cubic‐phase CdS after its combination with ReS_2_, suggesting the weak interaction formed via physical mixing at room temperature does not change the crystal structure.

**Figure 1:**
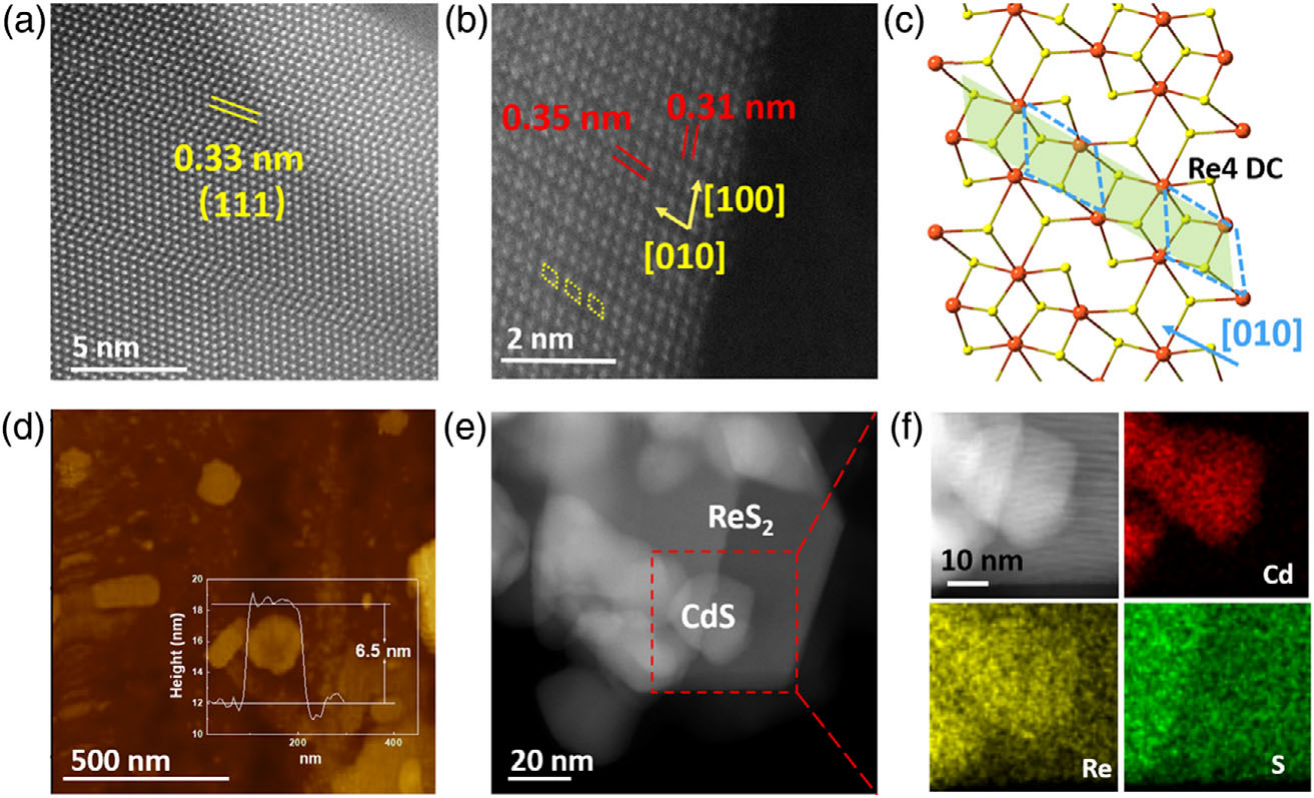
Aberration‐corrected HAADF‐STEM images of a) CdS NPs and b) ReS_2_ NSs. c) Simulated model for ReS_2_. The red and yellow colored spheres denote Re and S, respectively, and the blue‐dotted line denotes the Re_4_ diamond chain. d) AFM image of ReS_2_ NSs and measured thickness of ReS_2_. e) TEM of ReS_2_ NSs and CdS NPs. f) EDX mapping of CR12 from red‐dotted rectangle of (e).

Furthermore, the morphologies and microstructures of CdS NPs, ReS_2_ NSs, and CR12 are characterized by aberration‐corrected high‐angle annular dark field scanning transmission electron microscopy (HAADF‐STEM), transmission electron microscopy (TEM), and HAADF‐STEM elemental mapping. The HAADF‐STEM image of CdS NPs (**Figure** **1**a) exhibits a lattice spacing value of 0.33 nm, ascribed to the (111) facet of cubic‐phase CdS. In addition, the HAADF‐STEM image of a ReS_2_ NSs (Figure 1b) shows the typical Re_4_ diamond chain (DC) structure (yellow dot line square). The spacing values between the Re_4_ diamond in the *a* [100] and *b* [010] directions are 0.35 and 0.31 nm, respectively (Figure 1b). This is also displayed in the simulated atomic structure of the ReS_2_ monolayer (Figure 1c). It is reported that Re_4_ DCs can act as reactive sites for water splitting. This is because these favor water adsorption and activation, and possibly assist proton transfer to participate in CO_2_ photoreduction.^[^ ^33^, ^34^ ^]^ The thickness of the ReS_2_ NSs was further confirmed to be *≈*6.5 nm using atomic force microscopy (AFM; Figure 1d). The ultrathin thickness of the ReS_2_ NSs not only endows them with a large surface area to form strong electronic coupling with other materials, but also increases the number of exposed active sites toward catalytic reactions. The combination of a ReS_2_ NSs and CdS NPs in CR12 is shown in Figure 1e. Moreover, the HAADF‐STEM image and the corresponding energy dispersive X‐ray (EDX) elemental mapping images further corroborate the hybridization of the ReS_2_ NSs and CdS NPs (Figure 1f) in CR12. The colors red, yellow, and green in Figure 1f represent, respectively, the distribution of Cd, S, and Re elements, in agreement with the HAADF‐STEM image.

**Figure 2:**
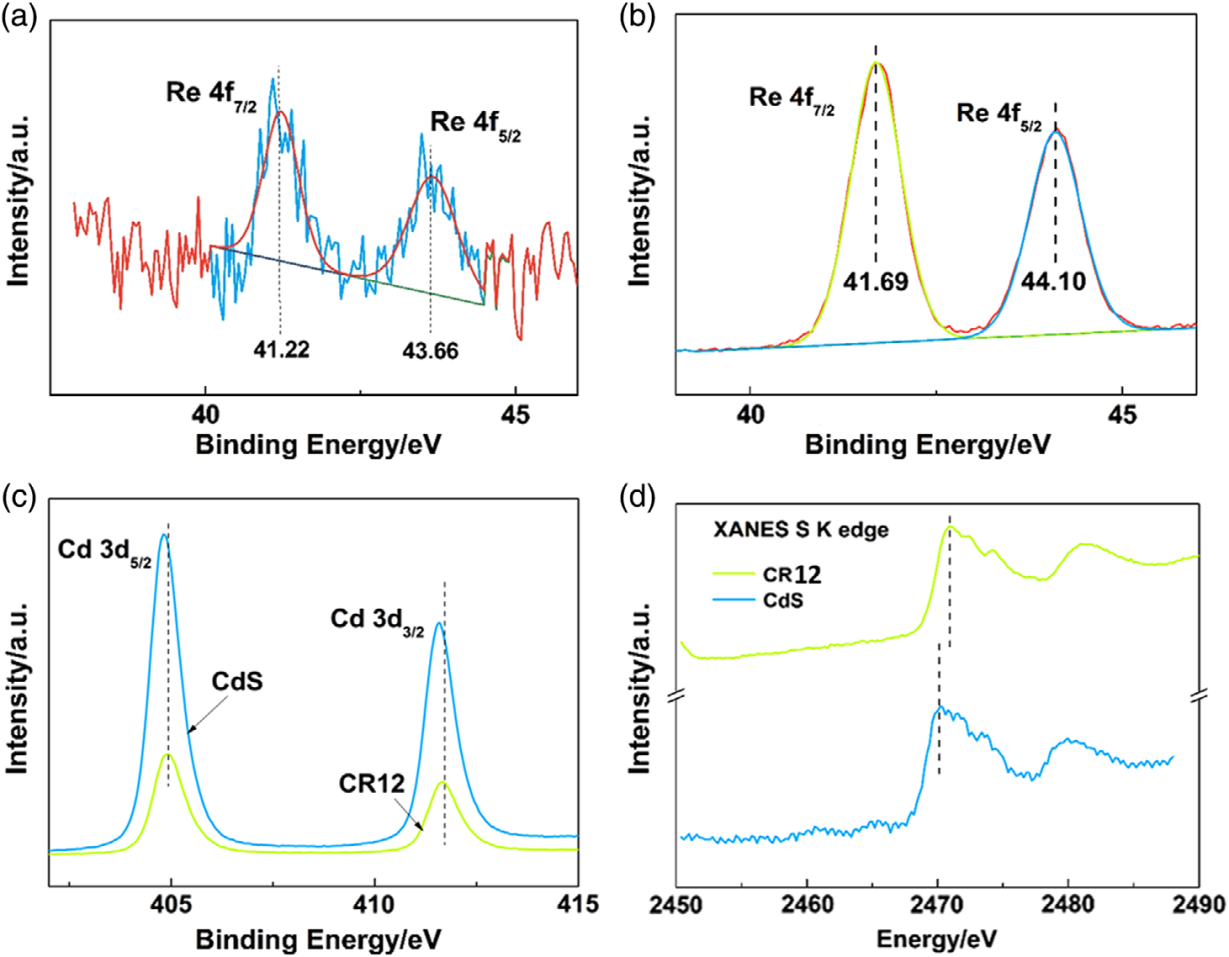
a) High‐resolution XPS spectrum of Re 4*f* for CR12. b) High‐resolution XPS spectrum of Re 4*f* for ReS_2_. c) High‐resolution XPS spectrum of Cd 3*d* for CdS and CR12. d) XANES S K edge of CdS and CR12.

Moreover, both surface‐sensitive XPS and synchrotron‐based XANES were conducted to disclose the interactions between CdS and ReS_2_ in CR12. The high‐resolution Re 4*f* XPS spectrum for CR12 (**Figure** **2**a) showed a shift of *≈*0.5 eV toward the direction of lower binding energy compared with that for pure ReS_2_ (Figure 2b), suggesting electron transfer from CdS to ReS_2_ in CR12. In addition, the high‐resolution XPS spectrum of Cd 3* d* for CR12 shifts to the direction of higher binding energy (Figure 2c), also indicating the electron migration from CdS to ReS_2_ in CR12. Furthermore, synchrotron‐based XANES was performed (Figure 2d). The S K‐edge XANES of CR12 indicates a shift toward high photon energy direction, in comparison to that for CdS. This also supports the electron transfer from CdS to ReS_2_ in CR12.

**Figure 3:**
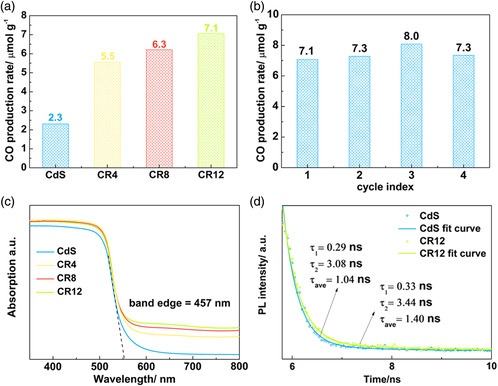
a) Photocatalytic CO_2_ reduction activities of CdS, CR4, CR8, and CR12 under visible‐light illumination (*λ* ≥ 420 nm). b) Photocatalytic CO_2_ reduction activity of CR12 recycle test with 7 h per cycle test. c) UV–vis diffuse reflectance spectroscopy and band edge for CdS, CR4, CR8, and CR12. d) TSPL spectra for CdS and CR12.

Solid–gas phase photocatalytic CO_2_ reduction activities of the as‐prepared samples were examined under visible‐light irradiation (*λ* ≥ 420 nm). As shown in **Figure** **3**a, CdS shows a limited photocatalytic CO_2_ reduction activity with CO and CH_4_ production of 2.3 and 1.1 μmol g^−1^ over 7 h (Figure S2, supporting information). The coupling of ReS_2_ and CdS (CR4, CR8, and CR12) leads to apparent enhancement of both activity and selectivity in visible‐light‐driven CO_2_‐to‐CO conversion (Figure 3a). In particular, CR12 shows the highest photocatalytic CO_2_‐to‐CO conversion activity of 7.1 μmol g^−1^ and selectivity 93.4%, *≈*309%, and 138% times higher than those of CdS alone. This result for the first time demonstrates that coupling with a ReS_2_ NS could significantly boost both activity and selectivity in photocatalytic CO_2_‐to‐CO conversion. In addition, we conducted three blank experiments under the same conditions but purged with ultra‐high‐purity argon gas instead of CO_2_, without visible‐light illumination and without a photocatalyst, respectively. These contrast experiments show no photocatalytic CO_2_ conversion performance, suggesting that the products (CO and CH_4_) are generated from photoinduced CO_2_ conversion. Furthermore, the stability of CR12 was studied via four‐cycle testing with 7 h per cycle (Figure 3b). No apparent deterioration in photocatalytic CO_2_ reduction performance of CR12 was found over a 28 h test. The TEM image and EDX spectrum (Figure S3, Supporting Information) of CR12 after photocatalytic CO_2_ reduction exhibited no obvious difference from those before reaction. This finding suggests that no apparent change in morphology and chemical composition of CR12 occurs after the 28 h photocatalytic reaction.

To further investigate the origin of the enhanced activity and selectivity, as well as the reaction mechanism in CR12 for photocatalytic CO_2_ reduction, both experimental characterizations and DFT‐based theoretical calculations were conducted. The light absorption capacity was studied by UV–vis diffuse reflectance spectroscopy. As shown in Figure 3c, increased absorption in the range of 460–800 nm is attributed to the presence of ReS_2_ NSs. However, the adsorption edge of CR12 (457 nm) does not display any shift. This finding suggests that the bandgap (2.24 eV) is not changed following combination with the ReS_2_ NSs. The bandgap of ReS_2_ NSs is 1.7 eV (Figure S4, Supporting Information). This induces the “bulge” at ≈600–800 nm in the UV–vis spectrum of CR12. Such an improved visible‐light absorption probably contributes to the raised activity in photocatalytic CO_2_ reduction.

A range of characterization methods, e.g., steady‐state photoluminescence (PL) spectroscopy, transient‐state photoluminescence (TSPL) spectroscopy, electrochemical impedance spectroscopy (EIS), and transient photocurrent (TPC) density measurement, were executed to probe the efficiency of charge‐carrier separation and transportation. The steady‐state PL (Figure S5, Supporting Information) intensity of CR12 is significantly lower than that of CdS. This finding is attributed to the oppressed charge‐carrier recombination after coupling with the ReS_2_ NSs in CR12. After fitting of the TSPL curves in Figure 3 d, elongated lifetimes of charge carriers (*τ*_1_ = 0.33 ns; *τ*_2_ = 3.44 ns; *τ*_ave_ = 1.40 ns) for CR12 were observed, in comparison to those for CdS (*τ*_1_ = 0.29 ns; *τ*_2_ = 3.08 ns; *τ*_ave_ = 1.04 ns). This also supports the more effective dissociation and migration of photogenerated electrons and holes in CR12. Furthermore, the EIS spectra (Figure S6a, Supporting Information) exhibit a smaller semicircle radius for the Nyquist plot together with a decreased charge‐transfer resistance (*R*_t_ = 4672 Ω) for CR12 in contrast with that of CdS (*R*_t_ = 4830 Ω). This finding suggests a faster charge‐carrier migration rate in CR12. In addition, CR12 exhibits a greater TPC density than CdS (Figure S6b, Supporting Information). This also confirms the more efficient dissociation of light‐induced excitons, in agreement with the earlier PL and EIS spectra results.

**Figure 4:**
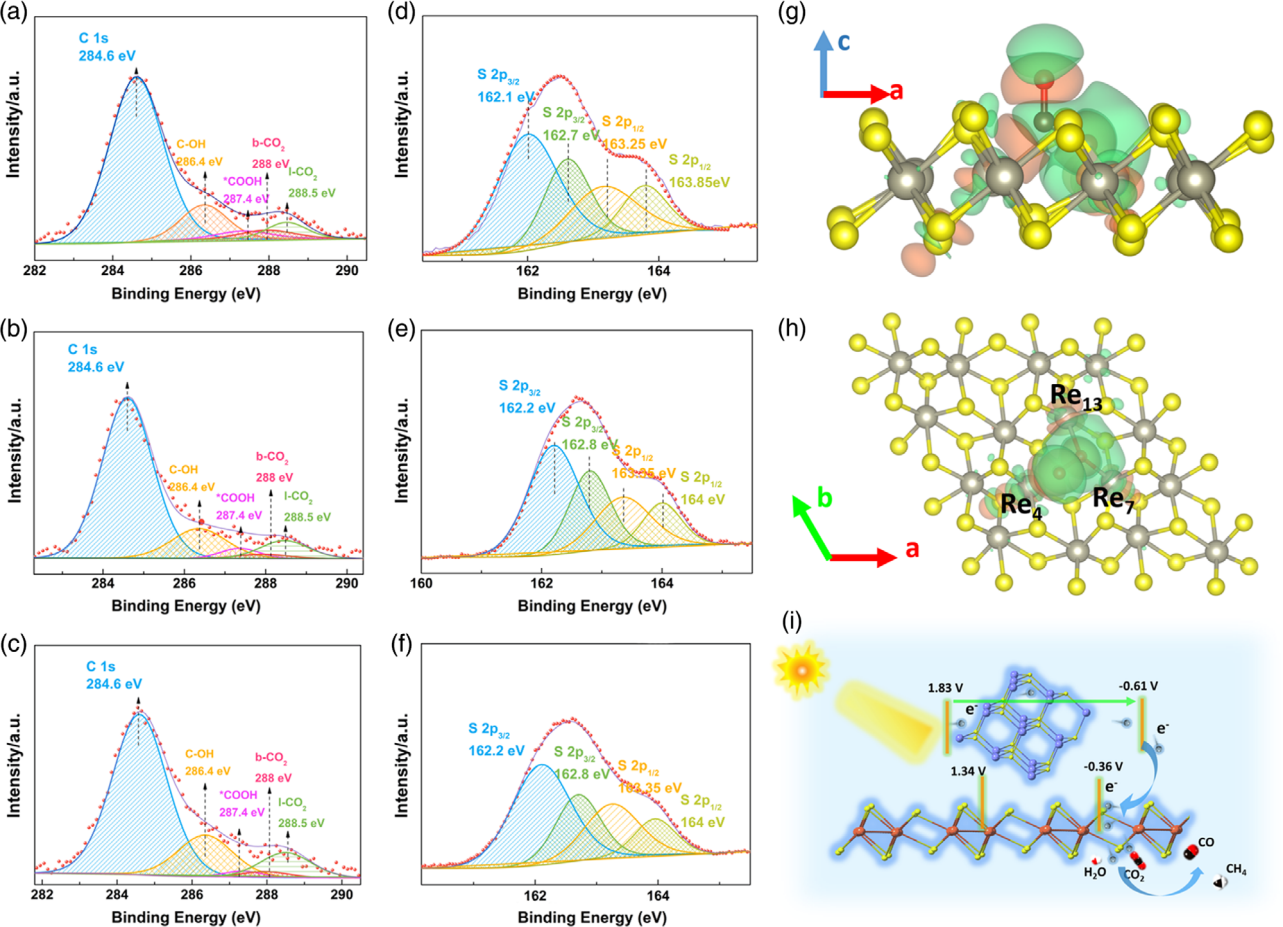
a,d) High‐resolution XPS spectrum for C 1*s* and S 2*p* for RS1. b,e) High‐resolution XPS spectrum for C 1*s* and S 2*p* for RS2. c,f) High‐resolution XPS spectrum for C 1*s* and S 2*p* for RS3. g) Side‐view (elevation) and h) top‐view (plan) of the electron density distribution of absorbed CO_2_ on V_s_‐ReS_2_. The red, yellow, gray, and brown colored spheres denote O, S, Re, and C atoms, respectively. The isosurface value is set to 0.002e Å^−3^. i) Schematic of photocatalytic CO_2_ reduction in CR12 system under visible‐light illumination (*λ* ≥ 420 nm). The purple, red, yellow, orange, white, and black colored spheres denote Cd, O, S, Re, H, and C atoms, respectively.

To investigate the CO_2_ adsorption, activation, and reduction process, XPS and Raman characterizations were conducted for pristine ReS_2_, RS1, RS2, and RS3 (see details in Section 1.5, Supporting Information). The C 1*s* peaks in **Figure** **4**a–c mainly consist of a peak located at 284.6 eV, ascribed to the contaminated carbon. All other peaks were calibrated using this peak. After deconvolution of the C 1*s* peaks in Figure 4a–c, four satellite peaks can be obtained and attributed to C—OH, *COOH, b‐CO_2_ (chemisorbed and bent CO_2_), and l‐CO_2_ (physisorbed and linear CO_2_), respectively.^[^ ^39^ ^]^ Among these adsorption configurations, *COOH is deemed as the precursor for CO, consistent with the major product (CO) of this work.^[^ ^40^, ^41^ ^]^ The contents (mol%) acquired based on the areas of four satellite C 1*s* peaks are provided in Table S1, Supporting Information). RS1 shows the highest contents of C 1*s* peaks associated with *COOH and b‐CO_2_, in comparison to RS2 and RS3. This is because the water vapor can facilitate the adsorption of CO_2_.^[^ ^42^, ^43^, ^44^ ^]^ The aforementioned results indicate that the adsorption of CO_2_ on the surface of the ReS_2_ NSs is obviously enhanced by visible‐light illumination. To further study the key role of illumination on CO_2_ adsorption, the high‐resolution XPS spectra of S 2*p* for pristine ReS_2_ NSs, RS1, RS2, and RS3 were collected. The ReS_2_ NSs exhibits two peaks located at 162.05 and 163.18 eV, ascribed to the S 2*p*_3/2_ and S 2*p*_1/2_ (Figure S7, Supporting Information). In comparison, after light illumination, satellite peaks located at 162.7 and 163.85 eV appear (Figure 4d–f), attributed to the presence of sulfur vacancy induced by visible‐light illumination. Moreover, the high‐resolution Re 4*f* XPS spectrum (Figure S7, Supporting Information) of the pristine ReS_2_ NSs only exhibits two peaks at 41.65 and 44.06 eV, attributed to the Re 4*f*_7/2_ and Re 4*f*_5/2_ peaks, respectively. In comparison, Re 4*f* satellite peaks located at lower binding energy positions are observed in the high‐resolution Re 4*f* XPS spectra of RS1, RS2, and RS3 (Figure S8a–c, Supporting Information), also implying the presence of sulfur vacancies in ReS_2_ after light illumination. Furthermore, the aberration‐corrected HAADF‐STEM images of RS1 and pristine ReS_2_ are displayed in Figure S9a,b, respectively. In contrast to pristine ReS_2_, RS1 exhibits more defects, e.g., vacancies and pores, on its surface after the light illumination. This finding is in agreement with the aforementioned XPS results.

These findings are further corroborated by the Raman spectroscopy characterization. Pristine ReS_2_ displays a typical *E*_g_‐like peak at *≈*305 cm^−1^ (Figure S10, Supporting Information). In contrast, RS1, RS2, and RS3 all exhibit a blueshift of the *E*_g_‐like peak. This might be caused by the presence of a sulfur vacancy leading to the mass fluctuation at the S position. In addition, RS1, RS2, and RS3 all exhibit peaks at *≈*1271 cm^−1^, ascribed to *COOH (Figure S11, Supporting Information).^[^ ^40^, ^41^ ^]^ In particular, the highest *COOH peak intensity is observed for RS1, compared to those of RS2 and RS3, in coincidence with the aforementioned XPS results (Table S1, Supporting Information). These findings were reinforced in the Fourier transform infrared spectroscopy (FTIR) spectra (Figure S12, Supporting Information). The bands at 1620 cm^−1^ are attributed to *COOH and the bands at 1404 cm^−1^ to the symmetric (O—C—O) vibration, indicating the presence of adsorbed CO_2_ molecules on the surface of the photocatalyst. These findings are consistent with those from the XPS results.

To verify the possible CO_2_ activation on the ReS_2_ with the sulfur vacancy (V_s_‐ReS_2_), DFT‐based theoretical computation was conducted to determine the adsorption of CO_2_ and the local electronic structure. As shown in Figure S13, Supporting Information, the atomic structures for V_s_‐ReS_2_ were constructed and optimized. The adsorption behavior of CO_2_ on the surface of V_s_‐ReS_2_ was studied (Figure 4g,h). The adsorption energy (*E*_ad_) of CO_2_ on V_s_‐ReS_2_ was found to be −0.82 eV. This value demonstrates that the chemisorption of CO_2_ on V_s_‐ReS_2_ is favorable.^[^ ^45^, ^46^ ^]^ The CO_2_ molecule loses linearity to change α(OCO) to 117.9°, and the two C—O_1_ and C—O_2_ bonds are lengthened to 1.209 and 1.398 Å, respectively, thereby denoting activation of the C=O bond on V_s_‐ReS_2_.^[^ ^46^, ^47^ ^]^

Bader charge analysis (Figure S14, Supporting Information) was conducted to study the charge distribution on the CO_2_‐adsorbed V_s_‐ReS_2_ and to investigate the electronic impact. It was found that Re atoms and C atoms are electron deficient, whereas S atoms and O atoms are negatively charged (Figure S14a, Supporting Information). In particular, Re near a sulfur vacancy (Re_4_, Re_7,_ and Re_13_) changed more positively when CO_2_ was absorbed onto V_s_‐ReS_2_ (Figure S14b, Supporting Information).^[^ ^38^, ^46^, ^47^ ^]^ Charge density difference was used to visualize the electron transfer behavior. The findings showed that Re and carbon atoms donate charges to oxygen and that they are positively charged.^[^ ^37^, ^46^, ^47^ ^]^ These findings confirm that V_s_‐ReS_2_ favorably impacts chemisorption and activation of CO_2_ and electron transfer between CO_2_ and V_s_‐ReS_2_.

Based on the findings from both the experimental studies and theoretical computations, a possible photocatalytic CO_2_ reduction mechanism is proposed. The conduction band (CB) and valence band (VB) edge positions of CdS and ReS_2_ are estimated via combining their Mott–Schottky plots (Figure S15, Supporting Information) and UV–vis diffuse reflectance spectrum (Figure S5, Supporting Information). As shown in Figure 4i, CdS and ReS_2_ form a type I (straddling‐type) heterojunction. Under visible‐light illumination (*λ* ≥ 420 nm), the CdS NPs are photoexcited and the electrons transfer from the VB to the CB, whereas the photogenerated holes in the VB of CdS will migrate to the VB of ReS_2_, where H_2_O molecules are oxidized. Then, photogenerated electrons in the CB of CdS transfer to the CB of ReS_2_. The ReS_2_ NSs accommodates abundant active sites for the adsorption, activation, and reduction of CO_2_ and H_2_O to evolve CO and CH_4_ (Equation ((1), (2))–(3)). The most likely pathways for the CO_2_ photoreduction on the CdS/ReS_2_ heterojunction are proposed as

(1) $$*\;+{\text{CO}}_{2}+e^{-}+H^{+}\to {\text{COOH}}^{*}$$

(2) $${\text{COOH}}^{*}+e^{-}+H^{+}\to {\text{CO}}^{*}+H_{2}O$$

(3) $${\text{CO}}^{*}\to *+\text{CO}↑$$

The asterisks and vertical arrows, respectively, denote reactive sites and the release of gas.

The presence of ReS_2_ NSs in CR12 leads to the apparent enhancement in the selectivity of photocatalytic CO_2_‐to‐CO conversion. This is probably because the electrons in ReS_2_ can be captured by tightly bound excitons to form trions consisting of two electrons and one hole,^[^ ^34^ ^]^ which facilitates the two‐electron reduction reaction of CO_2_‐to‐CO conversion rather than the eight‐electron reduction reaction of CH_4_ production.

## 3 Conclusions

In summary, we have successfully prepared a heterojunction of ReS_2_ NSs and CdS NPs using a facile self‐assembly method via physical mixing at room temperature. This nanocomposite exhibits a significantly boosted visible‐light photocatalytic CO production of 7.1 μmol g^−1^ together with an increased CO_2_‐to‐CO conversion selectivity of 93.4%. Such an improved photocatalytic performance originates from two factors: 1) intimate electronic interaction advancing efficient photogenerated electron–hole separation and migration and 2) in situ generated sulfur vacancies serving as active sites for CO_2_ adsorption, activation, and reduction to CO. These are verified by both state‐of‐the‐art characterizations, e.g., synchrotron‐based XANES, and theoretical calculations. Our work demonstrates the promising potency of ReS_2_ in light‐driven CO_2_ reduction and the intriguing opportunities of applying in situ generated anion vacancies of transitional metal dichalcogenides in catalysis, electronics, and optoelectronics.

## Conflict of Interest

The authors declare no conflict of interest.

## Supporting information

Supplementary Material
