# Supplementary material for: ReS2 Nanosheets with In Situ Formed Sulfur Vacancies for Efficient and Highly Selective Photocatalytic CO2 Reduction
Source: Small Sci. 2021 Jan 15;1(2):2000052. doi: 10.1002/smsc.202000052 (PMC11935916; doi:10.1002/smsc.202000052)
Supplement: Supplementary file 1 — Supplementary Material [file SMSC-1-2000052-s001.docx]

Supporting information

ReS_2_ Nanosheets with *in situ* Formed Sulphur Vacancies for Efficient and Highly-Selective Photocatalytic CO_2_ Reduction

Yanzhao Zhang, Dazhi Yao, Bingquan Xia, Haolan Xu, Youhong Tang, Kenneth Davey, Jingrun Ran,* and Shi-Zhang Qiao*

Y. Zhang, D. Yao, B. Xia, Dr. K. Davey, Dr. J. Ran, Prof. S. Z. Qiao

School of Chemical Engineering & Advanced Materials, The University of Adelaide, SA 5005, Australia.

E-mail: [s.qiao@adelaide.edu.au](mailto:s.qiao@adelaide.edu.au); [jingrun.ran@adelaide.edu.au](mailto:jingrun.ran@adelaide.edu.au)

Prof. H. Xu

Future Industries Institute, University of South Australia, SA 5095, Australia.

Prof. Y. Tang

Center for Nanoscale Science and Technology, School of Computer Science, Engineering, and Mathematics, Flinders University, SA 5042, Australia.

Part I: Experimental Section

# Materials and fabrication

*Fabrication of ultra-thin nanosheets of ReS_2_*: All reagents were analytic grade and used without further purification. Bulk ReS_2_ was purchased from Alfa Aesar. ReS_2_ nanosheets were prepared by a liquid-phase exfoliation using an ultrasonic probe followed by ice-bath sonication in deionized water. Bulk ReS_2_ (50 mg) was dispersed in deionized water for 2 h. The ultrasonic probe was operated 2 s and stopped for 4 s. Subsequently, the dispersion was centrifuged at 8000 rpm for 2 mins to collect supernatant. The concentration of the supernatant was determined to be 50 μg mL^-1^. This was confirmed by inductively coupled plasma mass spectrometry using a Perkin Elmer Nexion 350 D.

*Fabrication of CdS nanoparticles*: Nanoparticles were prepared by a precipitation-hydrothermal method. 3.424g Cd(NO_3_)_2_·4H_2_O was added into 87 mL deionized water followed by stirring for 60 min. Subsequently, 20 mL 0.9 M Na_2_S aqueous solution was added dropwise into the solution, followed by stirring for 1 h. The suspension was transferred to a 200 mL Teflon-lined autoclave and kept at 180 °C for 12 h. The final products were washed by deionized water and ethanol twice and dried at 60 °C for 5 h.

*Fabrication of CdS/ReS_2_ heterojunction:* ReS_2_ coupled CdS was fabricated by mechanically mixing the ReS_2_ suspension with *as-fabricated* CdS in an agate-mortar. 50 mg of *as-fabricated* CdS was added to the agate mortar, followed by the addition of volume of ReS_2_ suspension. The suspension was mechanically ground for 15 mins. Water was evaporated naturally, and the remaining solid ground into powders as final product. The volume of ReS_2_ suspension was 0, 4, 8 and 12 mL and the resulting samples were labeled as CdS, CR4, CR8 and CR12, respectively.

# 1.2 Physicochemical characterization

X-ray diffraction (XRD) patterns were recorded on a powder X-ray diffractometer (Miniflex, Rigaku) using Cu Kα radiation. Transmission electron microscopy under STEM mode (FEI Titan Thermis, 200 kV) was utilized to obtain HRTEM EDX mapping. XPS measurement was performed on a VGESCALAB 210 XPS spectrometer with Mg Kα source. The binding energies were referenced to the C 1s peak at 284.8 eV. The XANES measurements were performed in an ultra-high vacuum chamber of the undulator soft X-ray spectroscopy beamline at the Australian Synchrotron. Samples were dispersed in deionized water and loaded and dried on Au-plates. The raw XANES data were normalized to the photoelectron current of the photon beam, measured on an Au-grid. UV S4 Visible diffuse reflectance spectra were obtained on a UV-Vis spectrophotometer (UV2600, Shimadzu, Japan). An RF-5301PC spectrofluorophotometer (Shimadzu, Japan) was employed to obtain steady-state photoluminescence (PL) spectra at room temperature (25 ℃). The transient-state PL decay curves were obtained on an FLS1000 fluorescence lifetime spectrophotometer (Edinburgh Instruments, UK). Raman spectra were acquired by an iHR550 Raman microscope (HORIBA Scientific) with a charge-coupled device (CCD) detector and, a confocal microscope. Fourier Transform Infrared Spectroscopy was collected from the Nicolet 6700.

# 1.3 Photocatalytic CO_2_ reduction test

The photocatalytic CO_2_ reduction experiments were conducted in a 287 mL home-made reactor, sealed with silicone-rubber septa at ambient conditions. A 300 W Xenon arc lamp equipped with a UV-cutoff filter (λ ≥ 420 nm) was employed as the light source to trigger the photocatalytic reaction. In a typical test 50 mg photocatalyst was dispersed in 10 mL deionized water and sonicated for 2 h. The photocatalyst was coated on the bottom of the reactor after the water was dried under infrared light. Before illumination the reactor was purged by ultra-pure wet CO_2_ gas bubbled from deionized water at flow rate of 40 mL for 1 h. Three blank experiments were conducted by the same process as that for CR12 but purged with ultrahigh purity argon instead of CO_2_, without visible-light illumination and without photocatalyst, respectively. The product was collected from the reactor and examined by a gas chromatograph (GC, 7890B, Agilent). The GC was fitted with Plot-Q and a 5Å sieve columns (Agilent) in series, TCD and methanizer/FID detectors, and UHP Ar (BOC) as the carrier gas.

# 1.4 Electrochemical and photoelectrochemical test

Mott-Schottky plots were obtained on an electrochemical analyser (CHI760D instruments) using 0.5 M Na_2_SO_4_ aqueous solution. The test was conducted in a standard, three-electrode system with the *as-fabricated* samples as the working electrode, a Pt wire as the counter electrode, and Ag/AgCl (saturated KCl) as a reference electrode. The alternating current (AC) frequency applied was 1200 Hz. In the identical three-electrode system the EIS measurement was carried out in the range from 1 to 2 x 10^5^ Hz with an AC amplitude of 20 mV. 0.5 M Na_2_SO_4_ was employed as the electrolyte. The polarization curves were acquired in the three-electrode system. The bias sweep range was -1.5 to -0.8 V *vs.* Ag/AgCl with a step size of 5 mV. 0.5 M Na_2_SO_4_ was applied as the electrolyte. In the same three-electrode system the TPC response measurement was carried out. A 300 W Xenon light with a UV-cutoff filter (λ ≥ 420 nm) was utilized as the light source. 0.5 M Na_2_SO_4_ aqueous solution was applied as the electrolyte. The working electrodes were prepared as follows: 10 mg sample, 15 mg polyethylene glycol (PEG; molecular weight: 20000), and 1.0 mL ethanol were ground together to make a slurry. A doctor-blade method was used to coat the slurry onto a 2 x 1.5, cm FTO glass electrode. Under flowing N_2_ the acquired electrode was dried and heated at 350 °C for 0.5 h.

# 1.5 XPS and Raman testing of CO_2_ adsorbed ReS_2_

To investigate the adsorption of CO_2_ on the ReS_2_ surface three samples were prepared under different conditions that employed XPS and Raman to study surface chemical composition of the ReS_2_. ReS_2_ nanosheets were loaded on the sealed reactor. Ultra-high purity argon was purged into the reactor for 40 min. The reactor was under Xenon lamp illumination (with a UV cut off filter, λ ≥ 420 nm) for 14 h. Following Xenon lamp illumination ultra-high purity CO_2_ gas with water vapor was purged into the reactor for 5 h to reach the [adsorption](javascript:;) [equilibrium](javascript:;). ReS_2_ was collected and denoted as RS1. The second sample was prepared by the same process as that for RS1 but purged with CO_2_ without water vapor. It is denoted as RS2. The third sample was prepared with the same process as the RS2 with the Xenon lamp illumination lasting for 28 h. It is labeled as RS3.

Part II: Computation Section

# 1.1 Computational parameters and models

Computational models for ReS_2_ and sulphur vacancy containing ReS_2_ (V_s_-ReS_2_) were built according to the characterization results and reports in literature.^[1, 2]^ The Vienna *ab initio* Simulation Package (VASP) was used in DFT computations. The projector augmented wave (PAW) method with generalized gradient approximation (GGA) proposed by Perdew, Burke, and Ernzerh (PBE) was performed. The ReS_2_ monolayer with 4 x 4 supercell and CO_2_ adsorbed V_s_-ReS_2_ contained 16 Re and 31 S, 1 C atom, and 2 O atoms (shown in Figure S11 supporting information). The vacuum layer in the perpendicular direction was set as 20 Å.^[1]^ The cut-off energy for plane wave expansion was set at 450 eV. The convergence criterion for electronic structure iteration was set at 10^–5^ eV. For geometry optimization the structures were relaxed to forces on all atoms smaller than 0.02 eV/Å. A Gaussian smearing of 0.20 eV was applied during the geometry optimization and for total energy computations. For the energy computations the Brillouin zone was sampled using Gamma centered-grids for all structures and K-points set at 2 x 2 x 1. The DFT-D2 method of Grimme was employed in all computations to address van der Waals (vdW) interactions between atoms.^[3]^ Adsorption energy (*E_ad_*) is equal to, *E_ad_= E_total_ – E*CO_2_ *– E_substrate_*, where *E_total_* for the total energy of V_s_-ReS_2_ with one CO_2_ molecule adsorbed, *E*CO_2_ for thermodynamic energy of CO_2_ gaseous molecule and the *E_substrate_* for the energy of the initial system (V_s_-ReS_2_).^[1]^ Charge difference (*ρ_diff_*) was computed according to *ρ_diff_ = ρ_total_ – ρ*CO_2_*_*_ – ρ_substrate_*, where *ρ_total_* for total charge density of V_s_-ReS_2_ with one CO_2_ molecule adsorbed, *ρ*CO_2_*_*_* and *ρ_substrate_* for charge density of CO_2_ gaseous molecule and the initial system (V_s_-ReS_2_) respectively.^[4]^

Part III: Supplementary Results


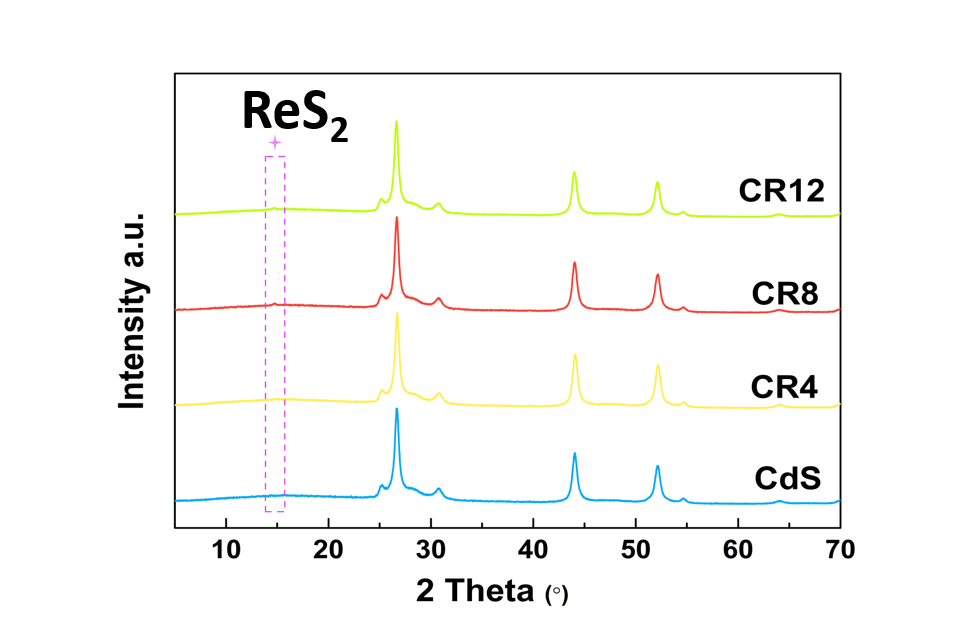


**Figure S1.** XRD patterns for CdS, CR4, CR8 and CR12. The pink-colored cross and purple circle, denote peaks of ReS_2_ and cubic CdS, respectively.


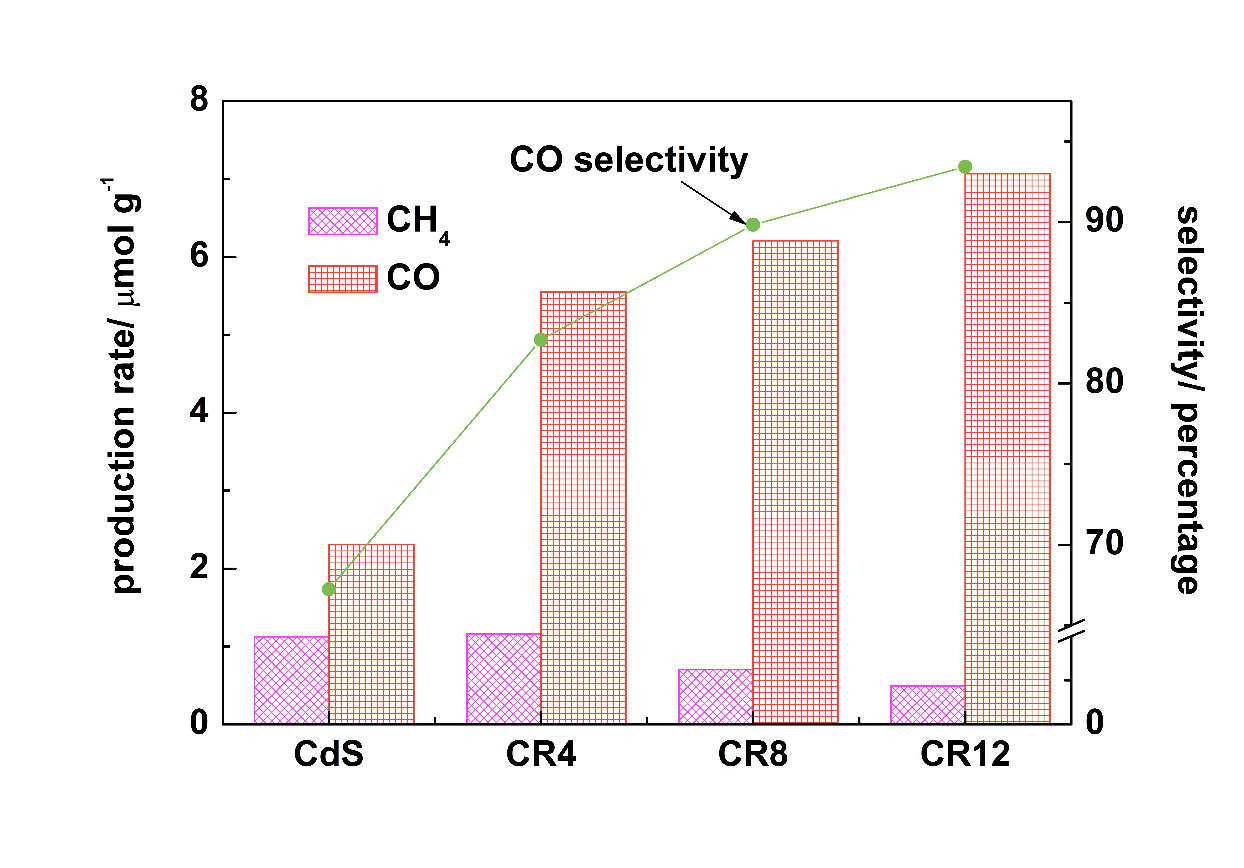


**Figure S2.** CO and CH_4_ production of photocatalytic CO_2_ reduction of CdS, CR4, CR8 and CR12 under visible-light illumination (λ > 420 nm) and corresponding CO selectivity.


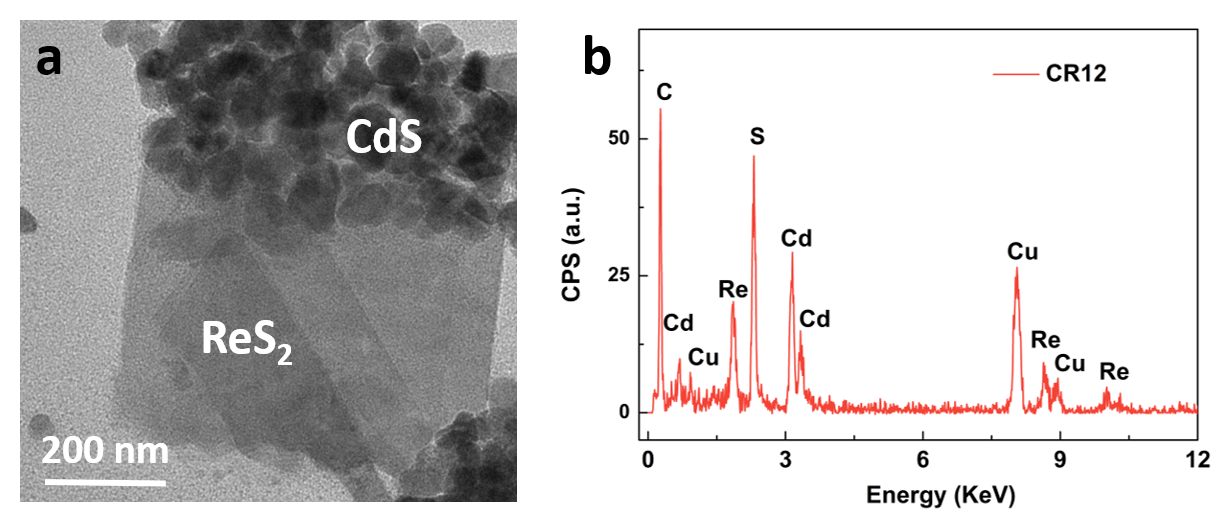


**Figure S3.** a) TEM image of CR12 after photocatalytic CO_2_ reduction testing and b) corresponding EDX spectrum.


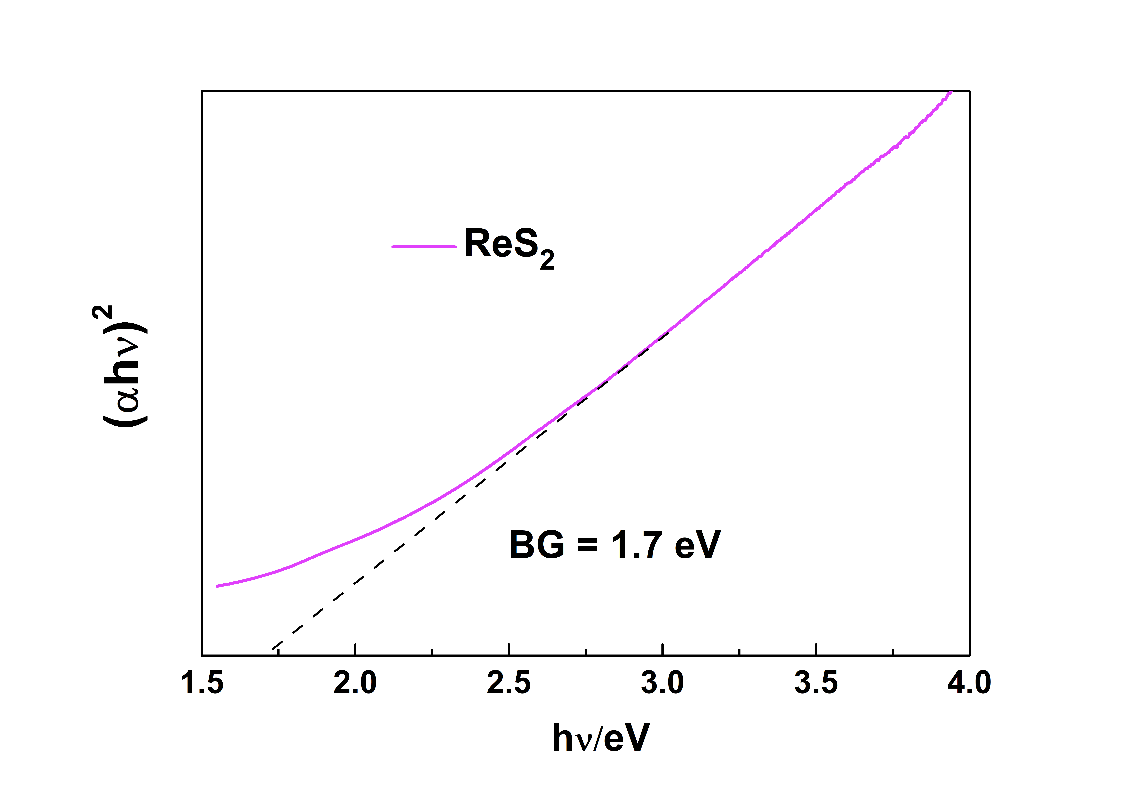


**Figure S4.** Tauc plot of ReS_2_ NSs and bandgap.


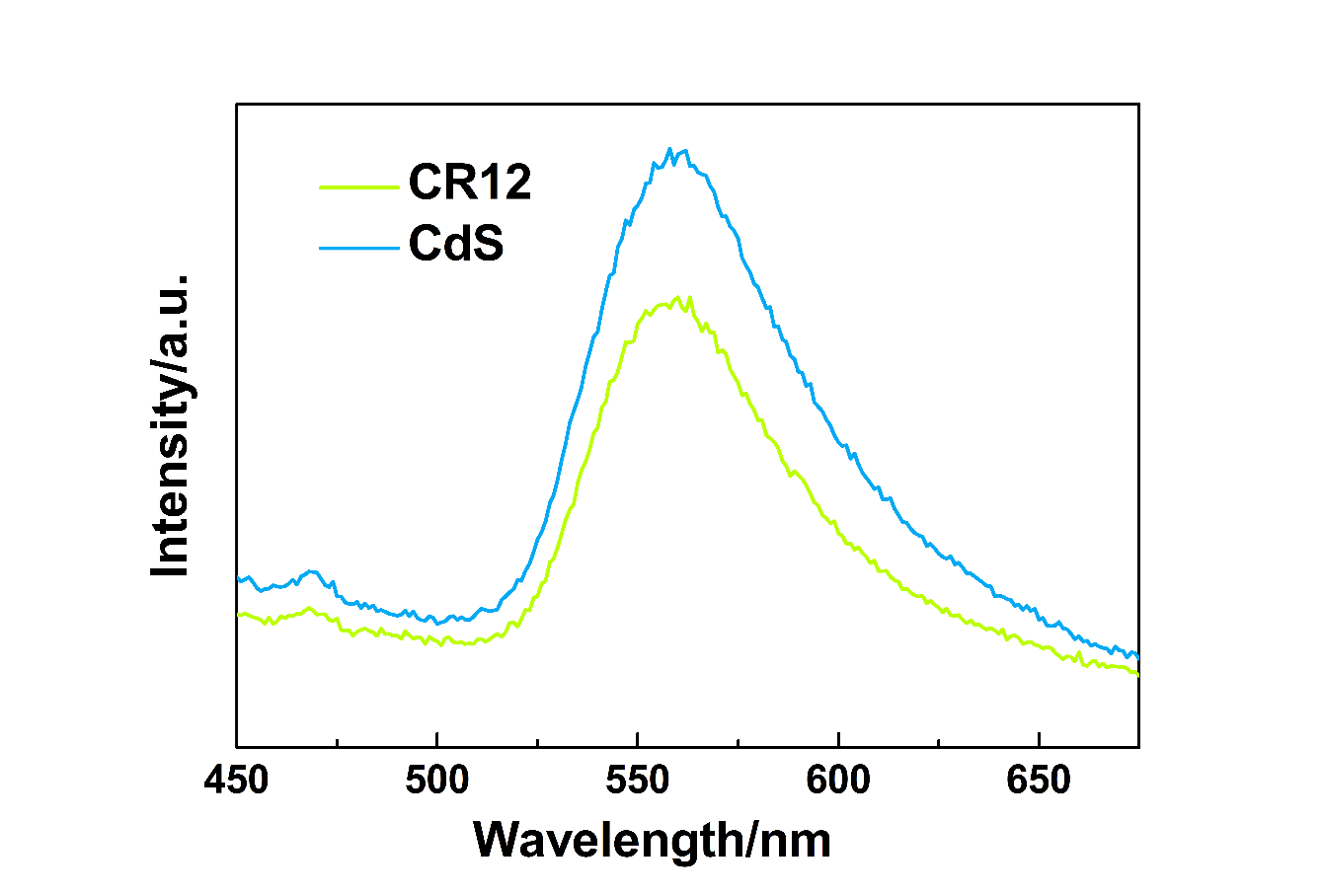


**Figure S5.** Steady-state photoluminescence (PL) spectra for CdS and CR12.


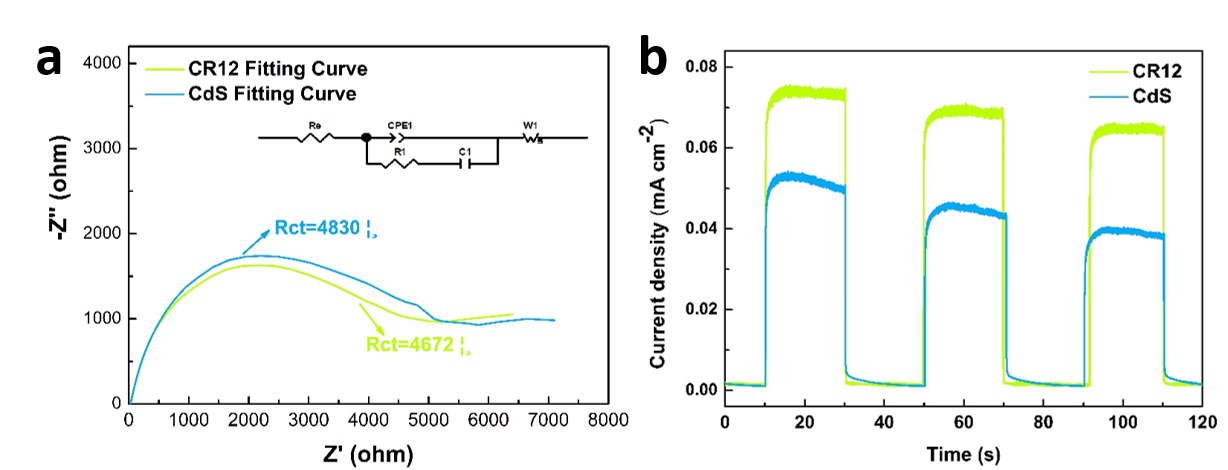


**Figure S6.** a) EIS Nyquist plots of CdS and CR12 in 0.5 M Na_2_SO_4_ aqueous solution. b) Transient photocurrent density measurement for CdS and CR12 in 0.5 M Na_2_SO_4_ aqueous solution.


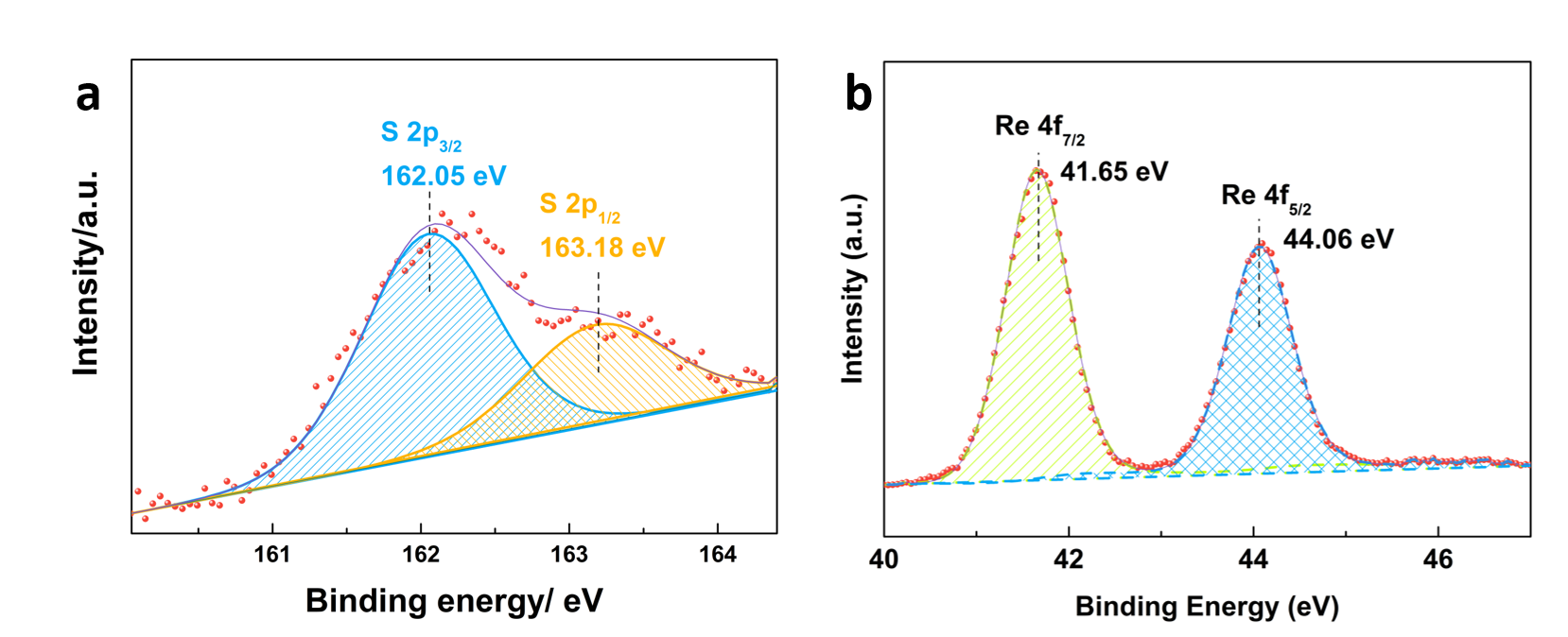


**Figure S7.** High-resolution Re 4f and S 2p XPS spectrum of ReS_2_.


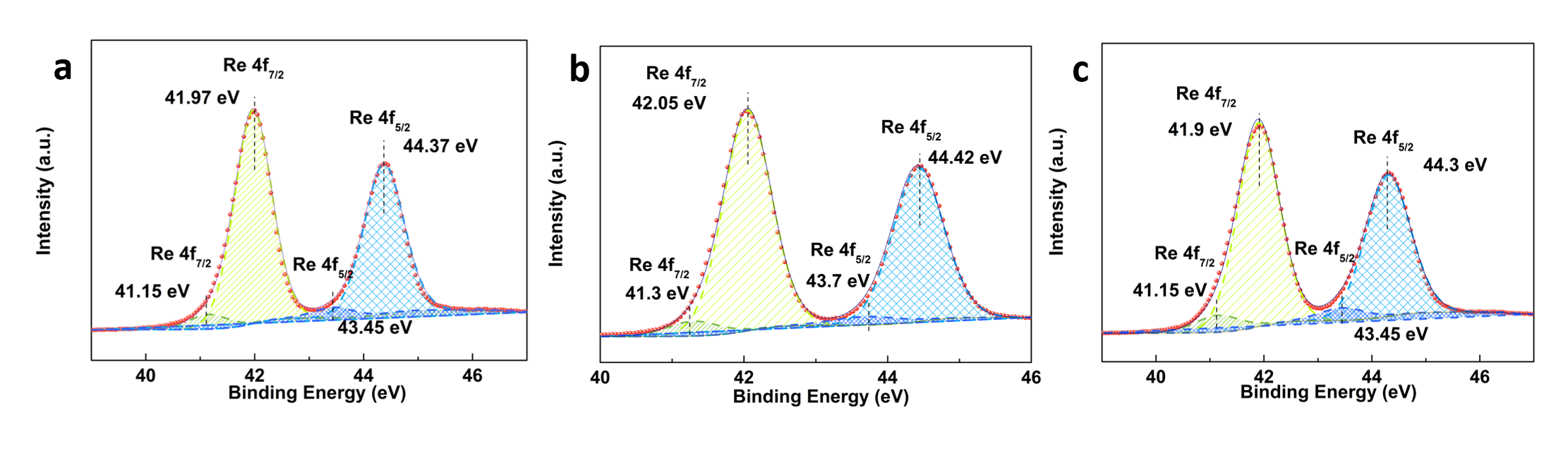


**Figure S8.** High-resolution Re 4f XPS spectrum of RS1, RS2 and RS3.


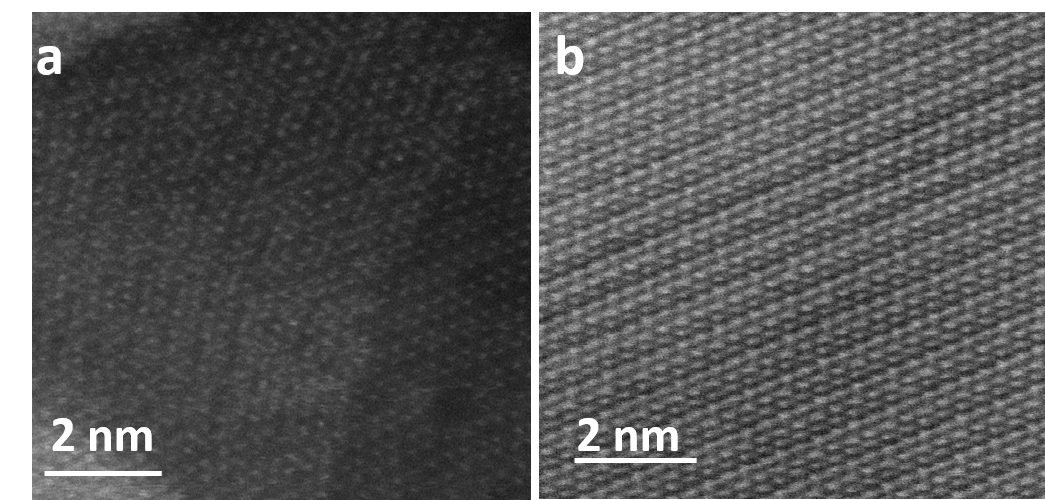


**Figure S9.** HRTEM for RS1 (a) and ReS_2_ (b).


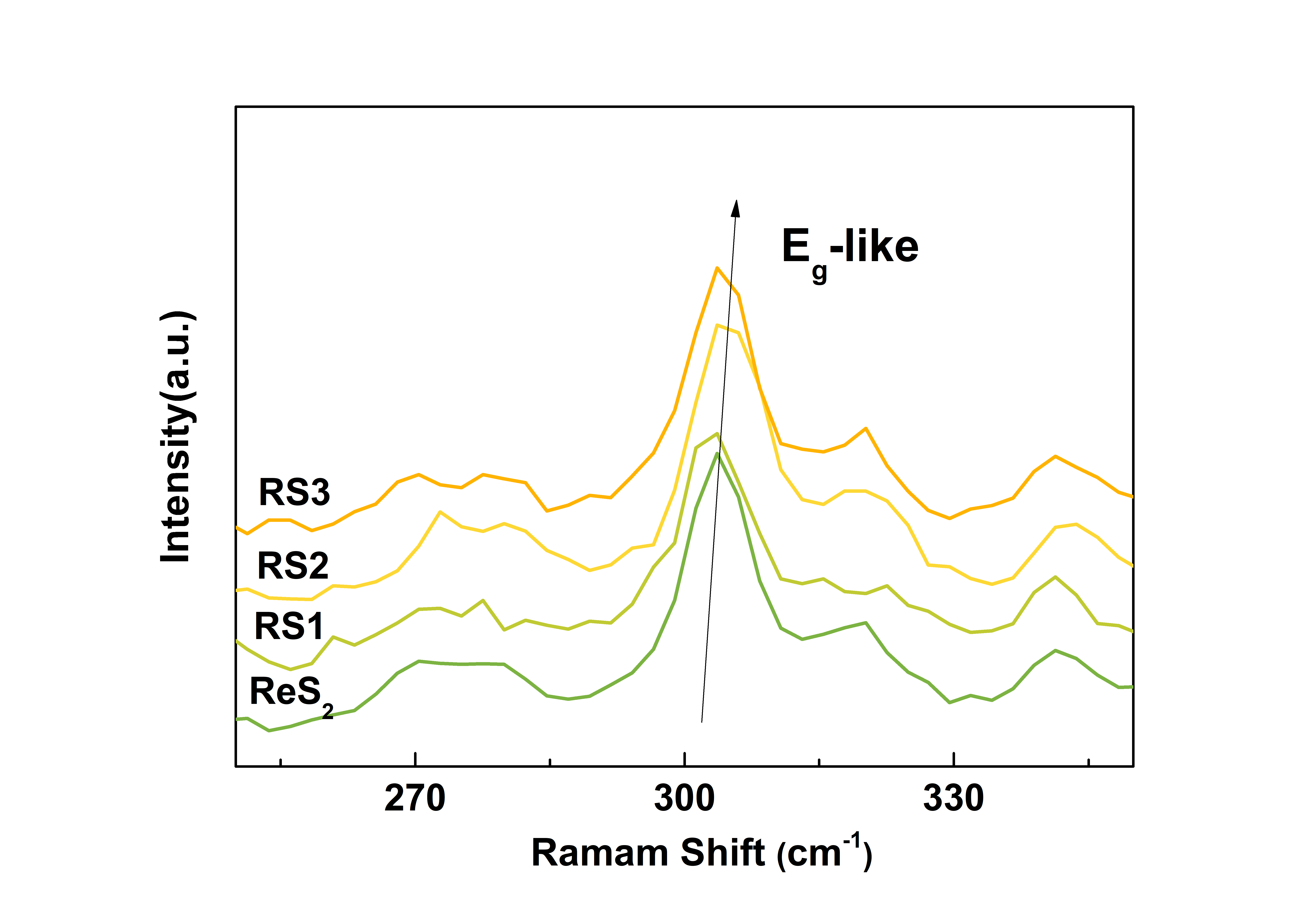


**Figure S10.** Raman spectra for E_g_-like peaks of pristine ReS_2_, RS1, RS2 and RS3.


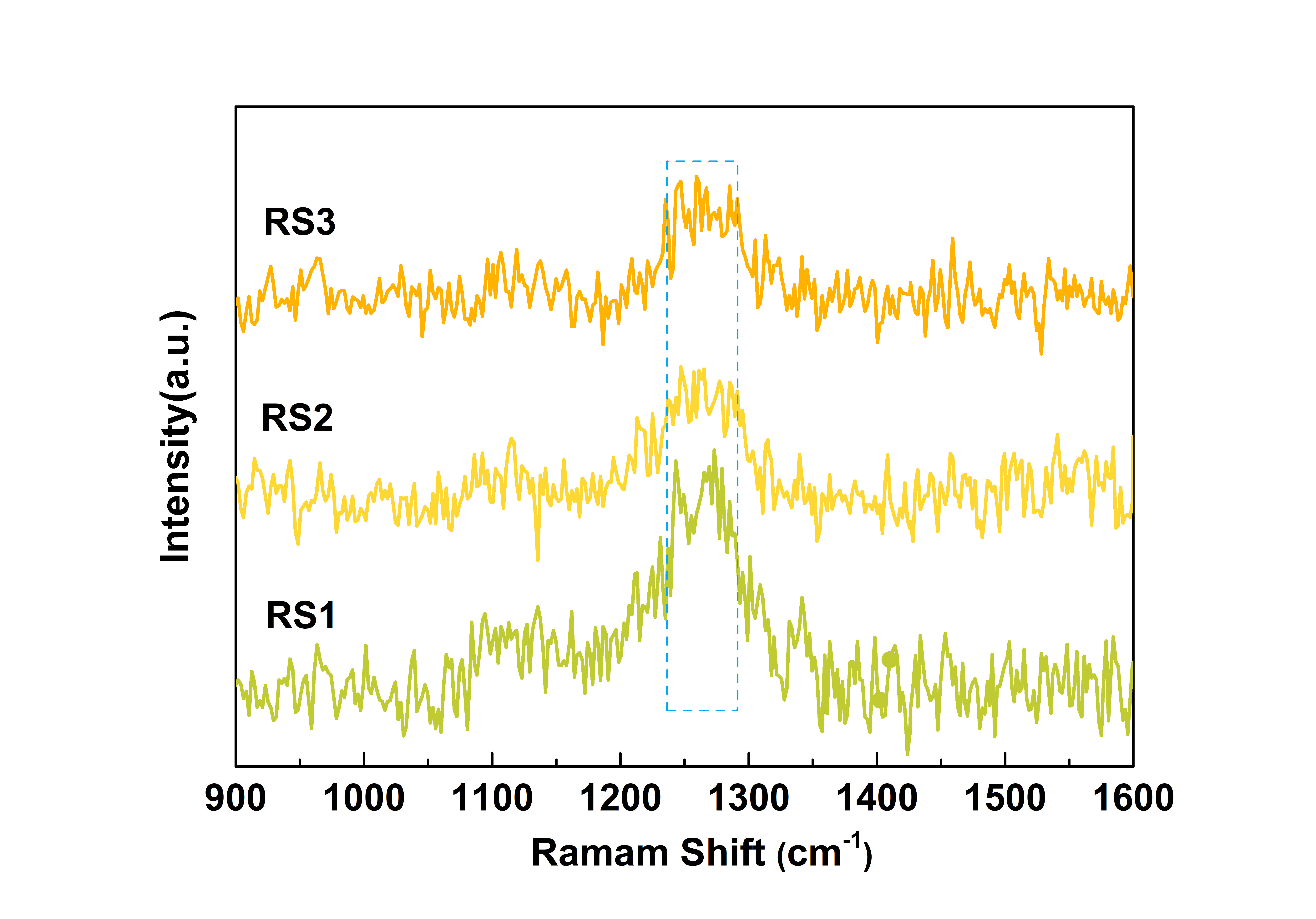


**F****igure S11.** Raman spectra for RS1, RS2 and RS3 with CO_2_ adsorption.


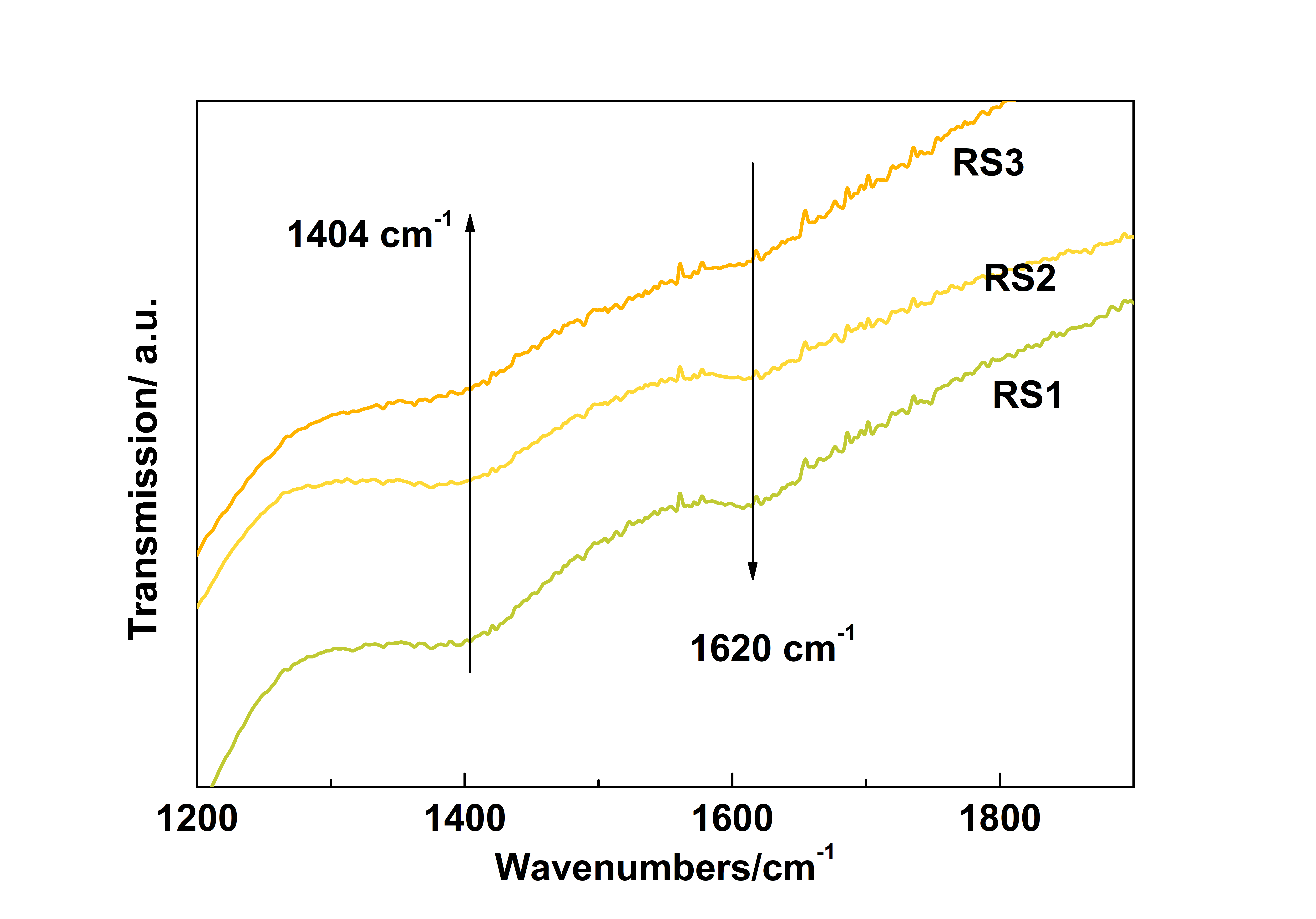


**Figure S12.** FTIR spectra for RS1, RS2 and RS3 with CO_2_ adsorption.


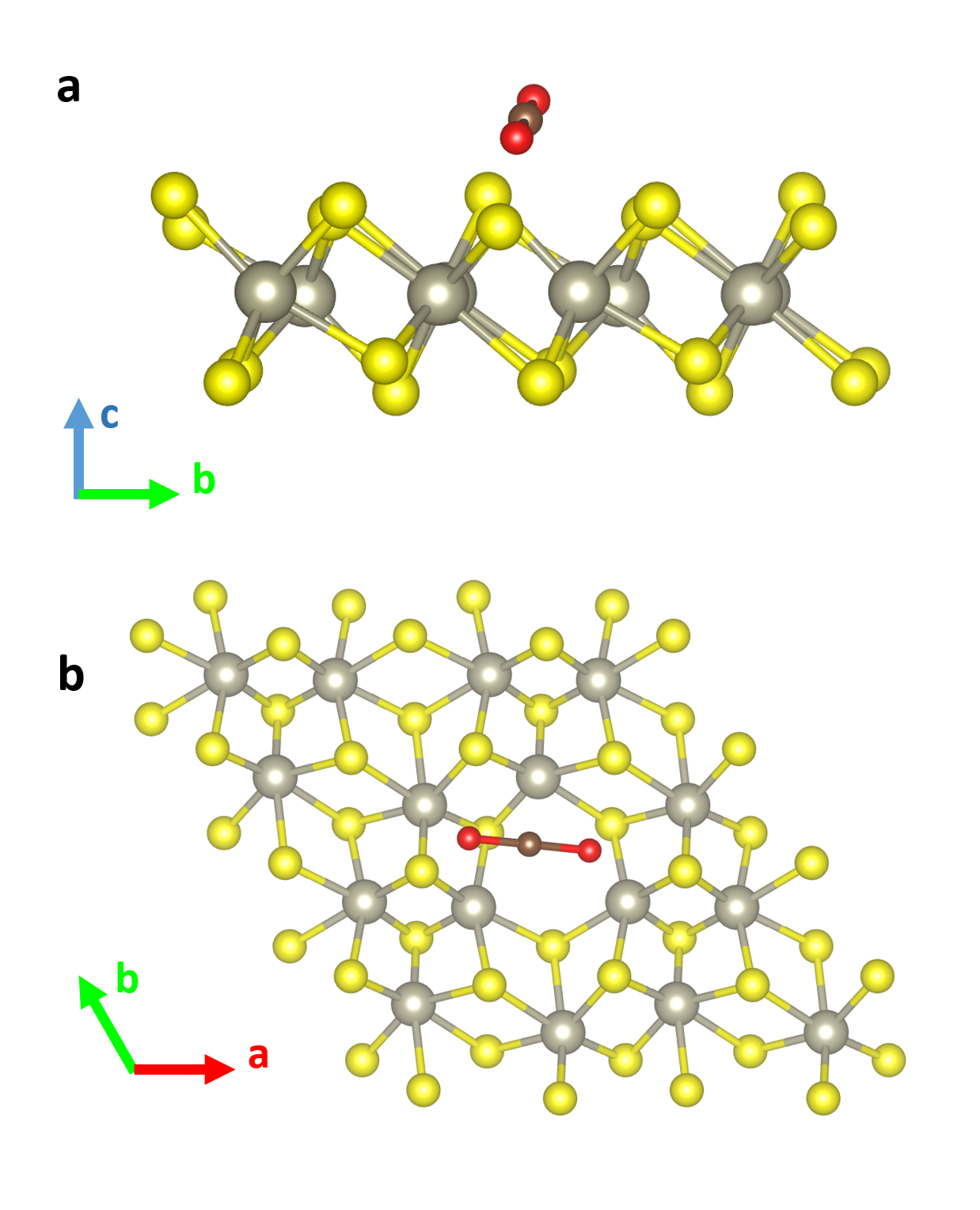


**Figure S13.** a) Side-view (elevation) and h) Top-view (plan) of CO_2_ molecule and atomic structures of Vs-ReS_2_. The red, yellow, grey and brown colored-spheres denote O, S, Re and C atoms, respectively.


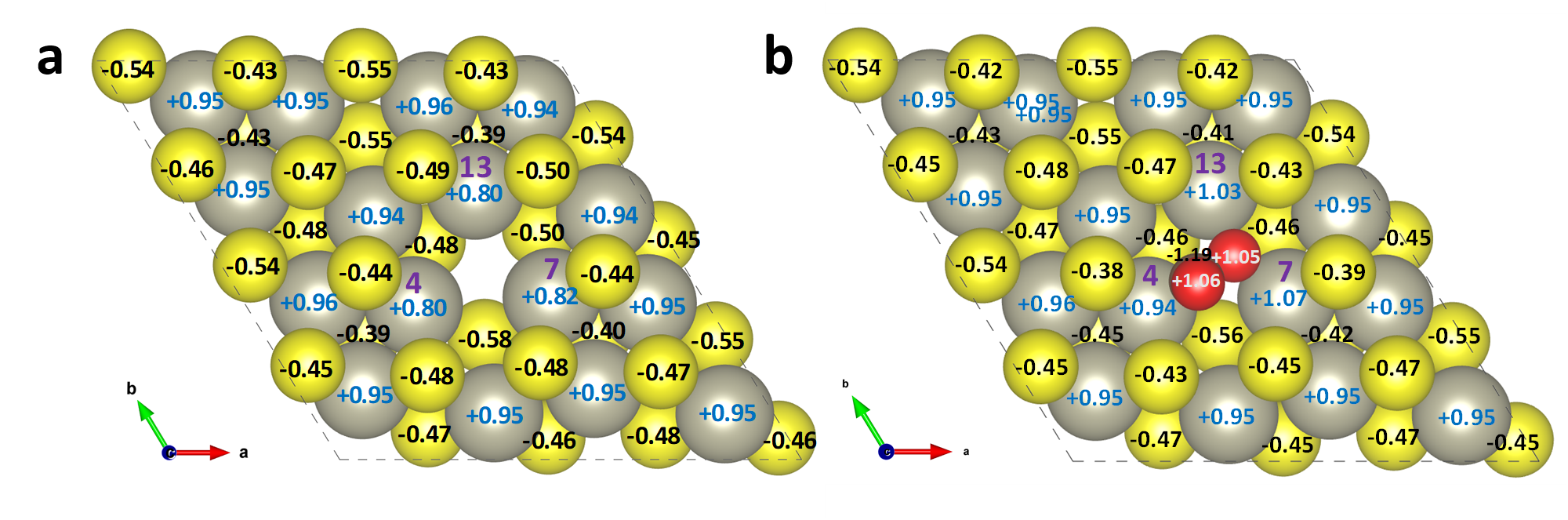


**Figure S14.** Bader charge analysis of a) V_s_-ReS_2_ and b) CO_2_ adsorbed on V_s_-ReS_2_. The yellow, grey, red and brown color-spheres represent Re, S, O and C atoms, respectively. All data are shown as two digits after the decimal. The positive and negative signs in the figure stand for, respectively, electron loss and accumulation that has a meaning similar to valency.


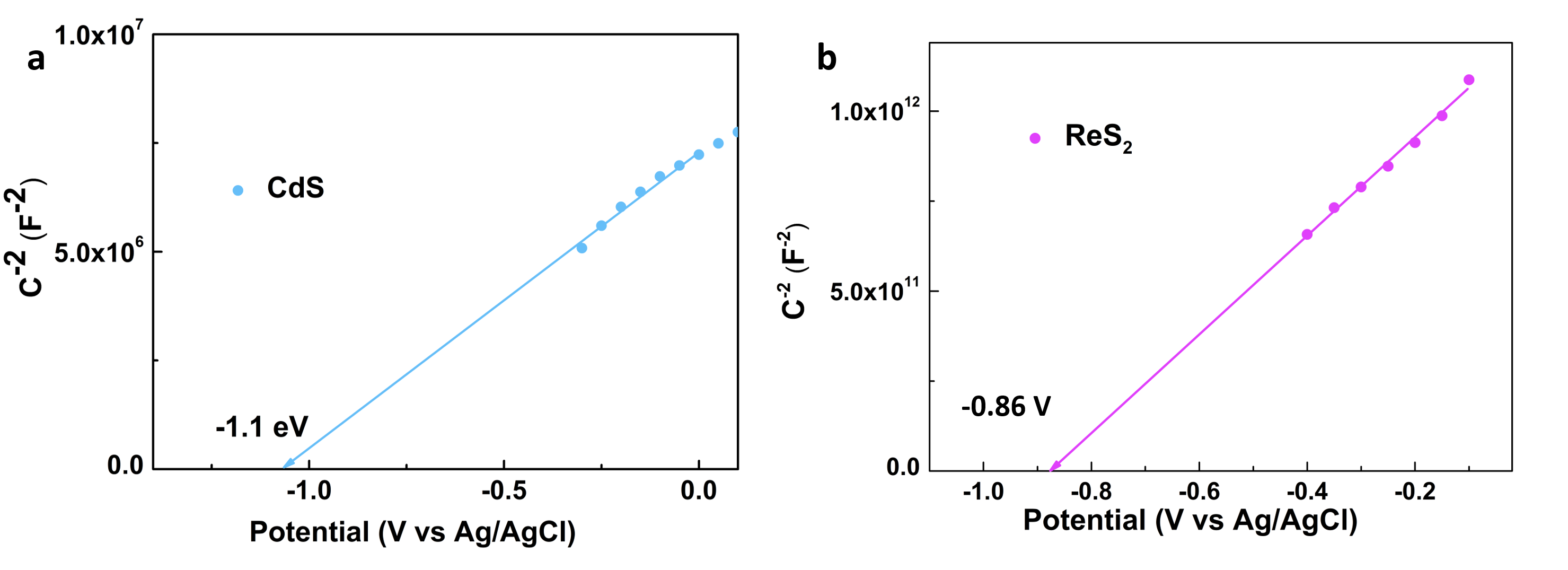


**Figure S15.** a) Mott-Schottky plot for CdS and b) ReS_2_ electrode in 0.5 M Na_2_SO_4_ aqueous solution. The plot suggests that the flat band potential for CdS (ReS_2_) is -1.1 V (-0.86 V) *vs.*Ag/AgCl, corresponding to -0.51 V (-0.26 V) *vs.* standard hydrogen electrode (SHE). The conduction band (CB) edge position of CdS (ReS_2_) is estimated therefore to be -0.61 V (-0.36 V) *vs.* SHE.

**Table S1.** Contents of surface carbon groups in ReS_2_, RS1, RS2 and RS3

| **Sample** | **Contents of carbon group from C 1s XPS**  **(mol%)** | | | |
| --- | --- | --- | --- | --- |
|  | C-OH | *COOH | b-CO_2_ | l-CO_2_ |
| RS1 | 13.38 | 3.68 | 3.99 | 5.45 |
| RS2 | 14.46 | 3.49 | 1.94 | 7.79 |
| RS3 | 16.74 | 3.35 | 2.18 | 8.7 |

**Part IV: Supplementary References**

[1] B. Chen, H. Li, H. Liu, X. Wang, F. Xie, Y. Deng, W. Hu, K. Davey, N. Zhao, S.Z. Qiao, *Adv. Energy Mater.* **2019**, *30*, 1970117.

[2] [J. Pan](https://www.sciencedirect.com/science/article/pii/S0375960119307029#!), [X. Zhou](https://www.sciencedirect.com/science/article/pii/S0375960119307029#!), [J. Zhong](https://www.sciencedirect.com/science/article/pii/S0375960119307029#!), [J. Hu](https://www.sciencedirect.com/science/article/pii/S0375960119307029#!), *Phys. Lett. A* **2019**, *31*, 125883.

[3] S. Grimme, *J. Comput. Chem.* **2004**, *25*, 1463.

[4] Y. Jiao, Y. Zheng, P. Chen, M. Jaroniec, S.Z. Qiao, *J. Am. Chem. Soc.* **2017**, *139*, 18093.
